# Laparoscopic attenuation of a congenital extrahepatic portosystemic shunt in a dog—a thin-film banding for splenophrenic shunt: A case report

**DOI:** 10.3389/fvets.2022.918153

**Published:** 2022-09-26

**Authors:** Jiyoung Park, Kwangsik Jang, Hyun Min Jo, Se Eun Kim

**Affiliations:** ^1^Department of Veterinary Surgery, Ulsan S Animal Medical Center, Ulsan, South Korea; ^2^Department of Veterinary Surgery, S Animal Cancer Center, Yangsan, South Korea; ^3^Department of Veterinary Surgery, College of Veterinary Medicine and BK21 Plus Project Team, Chonnam National University, Gwangju, South Korea; ^4^Preclinical Study Division, Biomaterial R&BD Center, Chonnam National University, Gwangju, South Korea

**Keywords:** extrahepatic portosystemic shunt, computed tomography angiography, laparoscopic attenuation, thin film banding, dog

## Abstract

A 6-year-old castrated male Shih-Tzu dog weighing 6. 5 kg presented with chief complaints of pollakiuria and urine dribbling. He had a history of urolithiasis for 3 years, which was confirmed by the presence of ammonium urate in the urinary stone analysis, performed 2 years prior to the presentation. Blood examination showed high values of fasting ammonia, post-prandial bile acid, and low blood urea nitrogen. Microhepatica and urolithiasis were identified on plain radiography and ultrasonography. A computed tomography angiography demonstrated a shunting vessel, diameter up to 9.6 mm, originated from the splenic vein, and linked with the phrenic vein. A surgical attenuation with a thin-film banding was performed under laparoscopic visualization. Left triangular ligament was incised, and one stay suture was placed to the stomach to expose the vessel. The shunting vessel was dissected before it entered the diaphragm, and a thin-film band was applied around the vessel. The patient recovered uneventfully without post-attenuation neurologic signs. Portal vein diameter increased with time, and complete closure of the shunting vessel was identified on computed tomography angiography performed at 14 months after attenuation. The patient was doing well for 31 months after surgery without protein restriction. This is a report of laparoscopic attenuation for splenophrenic type of canine congenital extrahepatic portosystemic shunt with a favorable outcome using thin-film banding.

## Introduction

Portosystemic shunt (PSS) is a condition of a vascular anomaly with direct communication between systemic and portal venous systems (1). It allows portal blood with toxins and hepatotropic agents to bypass the liver and to enter into the systemic circulation, which results in clinical manifestation of neurologic, gastrointestinal, or urological signs; vomiting, diarrhea, ammonium urate urolithiasis, stranguria, dysuria, hematuria, bacterial cystitis, hepatic encephalopathy, seizure, and reduced growth (1, 2). Owing to developmental error, a single congenital extrahepatic PSS (CEPSS) is most frequently diagnosed in young, purebred, small-breed dogs (3–5).

The eventual goal of the treatment in CEPSS is a complete occlusion of the shunting vessel to result in further restoration of normal hepatopetal blood flow, hepatic volume, and metabolic function (6). Surgery provides a superior survival rate and higher quality of life with a lower frequency of ongoing clinical signs compared to medical management only (7, 8). To avoid post-attenuation portal hypertension and the development of multiple acquired shunts, gradual attenuation techniques are commonly performed using the ameroid ring constrictor (ARC) or thin-film banding (TFB) with similarly favorable long-term outcomes in survival and post-attenuation seizures (3, 9). Preoperative medical management (antibiotics, antacids, antiseizure agents, synthetic disaccharide (lactulose), and a moderately protein-restricted diet) is recommended to stabilize patients with hepatic encephalopathy or to produce more suitable anesthetic candidates (6).

Surgical attenuation has been performed almost exclusively *via* celiotomy. In the literature, there are only two case reports available that describe laparoscopic approaches in canine CEPSSs (10, 11). This report describes a case of laparoscopic attenuation for splenophrenic shunt in a dog with a clinical course of preoperative diagnosis, detailed surgical procedure, and 31 months of follow-up, including post-attenuation computed tomography angiography (CTA).

## Case description

A 6-year-old castrated male Shih-Tzu dog, weighing 6.5 kg, was referred for an evaluation of PSS and further surgical treatment. He presented with a chief complaint of pollakiuria, urine dribbling, and vomiting. The patient had a 3-year history of urolithiasis confirmed by ammonium urate 2 years before presentation.

At the presentation, he was alert and in healthy condition with a good appetite and normal activity. Blood examinations (complete blood count, serum chemistry, and electrolytes) revealed normal values, except for fasting ammonia (72 μmol/L, reference range (RR), < 40 μmol/L) and blood urea nitrogen (BUN; 4 mg/dL; RR, 7–27 mg/dL). Coagulation parameters (PT, APTT) were in upper ranges, and pre- and post-prandial bile acid values were 7 μmol/L (RR, < 12 μmol/L) and > 30 μmol/L (RR, < 20 μmol/L), respectively (Table 1).

**Table 1:** Serum biochemical panel and portal vein diameter before and after laparoscopic attenuation of canine splenophrenic shunt.

**Parameter**	**Pre**	**3 month after LA**	**8 month after LA**	**14 month after LA**	**Reference range**
BUN (mg/dL)	4	8	10	8	7–27
Albumin (g/dL)	2.8	-	3.4	3.1	2.3–4.0
Cholesterol (mg/dL)	165	-	262	248	110–320
Glucose (mg/dL)	111	107	-	-	74–143
Fasting ammonia (μmol/L)	72	0	5	2	< 40
pre-prandial SBA (μmol/L)	7	< 5	13	18	< 12
post-prandial SBA (μmol/L)	> 30	30	> 30	28	< 20
Pv diameter (mm)	3	5.1	5.1	5.3	-
Pv/Ao ratio	0.42	0.72	0.72	0.75	-

LA, laparoscopic attenuation; BUN, blood urea nitrogen; SBA, serum bile acid; Pv, portal vein; Ao, aorta.

**Figure 1:**
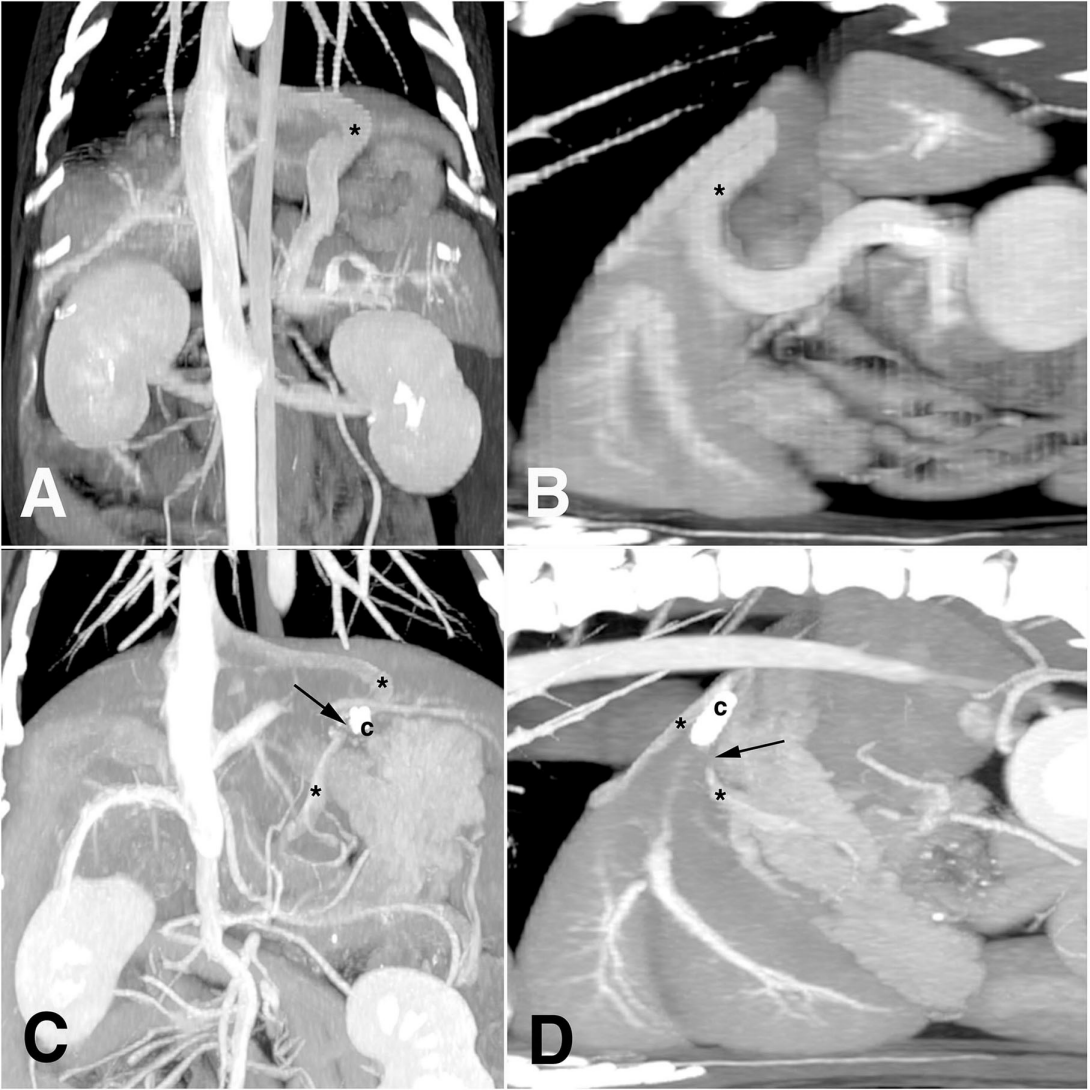
Diagnostic images of computed tomography angiography before **(A,B)** and after **(C,D)** laparoscopic attenuation of canine splenophrenic shunt using thin-film banding. Asterisk, shunting vessel; arrow, vascular gap of complete closure; c, ligation clip.

Plain thoracic and abdominal radiography showed microhepatica and multiple cystourethrolithiasis in low radio-opacity. On ultrasonography, a splenic nodule (8 mm in longest axis) and multiple urinary stones (up to 3 mm in diameter) were identified in both kidneys, urinary bladder, and penile/membranous urethra. The mean diameter of the portal vein at porta hepatis was 3 mm, and its ratio to the diameter of the aorta (7 mm) was 0.42 (Pv/Ao ratio). When a CTA with maximum intensity projection was performed, there was a shunting vessel, which originated from the splenic vein (Figures 1A,B). It ran cranially toward the diaphragm and was connected to post-hepatic caudal vena cava (CVC) between the liver and diaphragm. The diameter of the shunt was ~9.6 mm. A definitive diagnosis of single CEPSS (splenophrenic type) was made, and he was started on a low-protein diet and oral medications, including metronidazole (7.5 mg/kg), famotidine (0.5 mg/kg), ursodeoxycholic acid (5 mg/kg), S-adenosyl-L-methionine (17 mg/kg), and lactulose (0.5 ml/kg), twice a day. Urinary stones were moved to the bladder through retrograde urohydropropulsion under general anesthesia after CTA.

**Figure 2:**
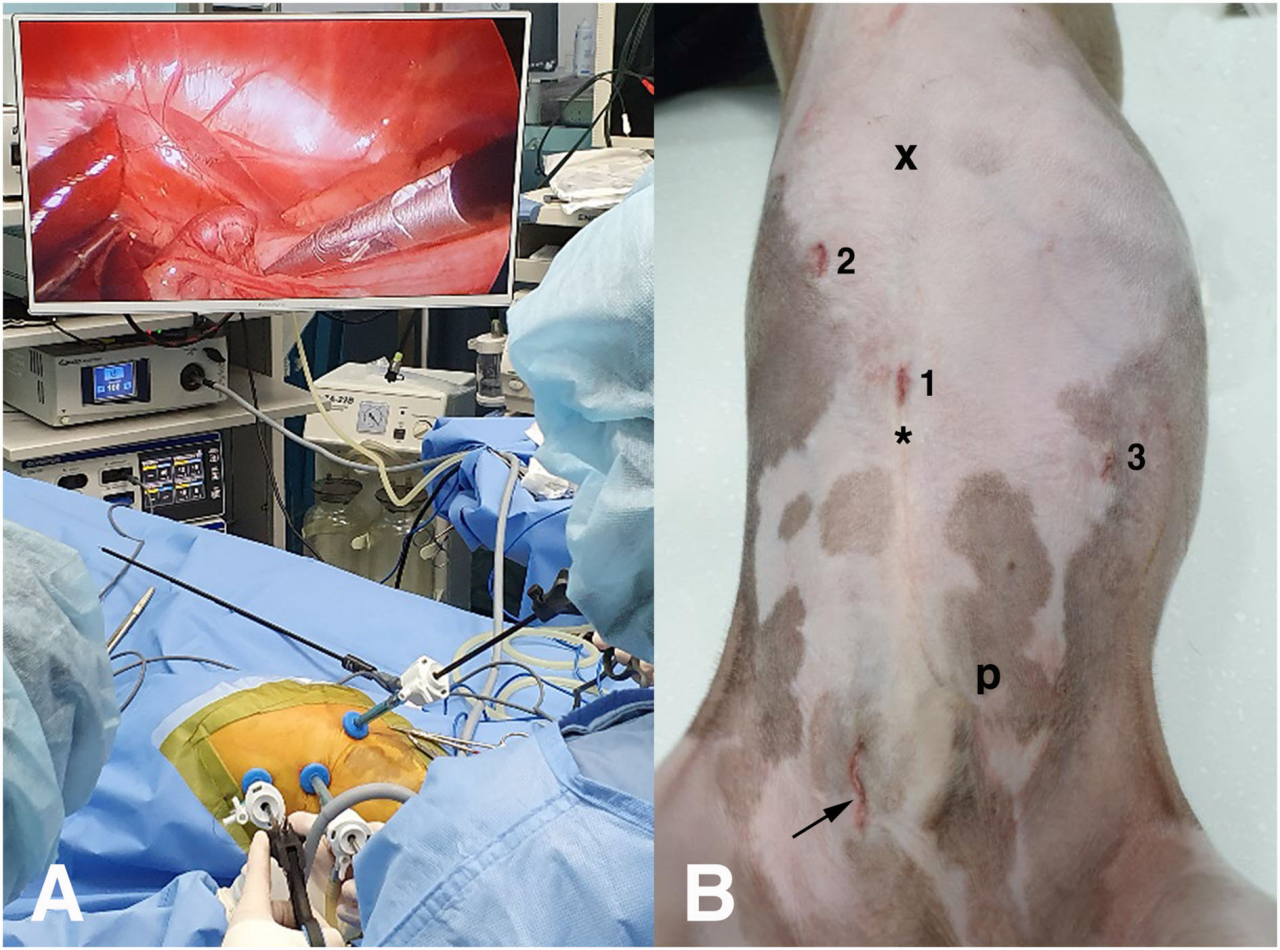
Laparoscopic tower set-up **(A)** and portal sites **(B)** for laparoscopic attenuation of canine splenophrenic shunt using thin-film banding. x, xiphoid process; 1, primary port; 2, second port; 3, third port; asterisk, umbilicus; arrow, mini-laparotomy site for cystolithotomy; p, prepuce.

Six weeks later, a laparoscopic PSS attenuation was performed under general anesthesia. Midazolam (0.2 mg/kg, IV), butorphanol (0.2 mg/kg, IV), propofol (4 mg/kg, slow IV), and isoflurane were used for premedication, induction, and maintenance. Cefazolin (20 mg/kg, IV) and meloxicam (0.2 mg/kg, IV) were also administrated. Oral levetiracetam (20 mg/kg, q8hr) was started 3 days before the surgery. The endoscopic tower was located rostral to the patient, while the surgeons stood on the opposite side (Figure 2A). The patient was in dorsal recumbency close to the end of the table. Three 5-mm ports were placed using the Hasson technique (Figure 2B) as follows: primary port (cranial to umbilicus) for the telescope (5 mm, 0°, 1488 HD; Stryker, Portage, Michigan, USA), second port (right paramedian near the costal arch), and third port (left paramedian caudal to the primary one) for laparoscopic surgical instruments. Intra-abdominal pressure was maintained under 11 mmHg throughout the procedure. The operating table was tilted to secure visualization putting the patient into the right oblique, reverse Trendelenburg, or supine position as needed according to the surgical steps during the procedure.

**Figure 3:**
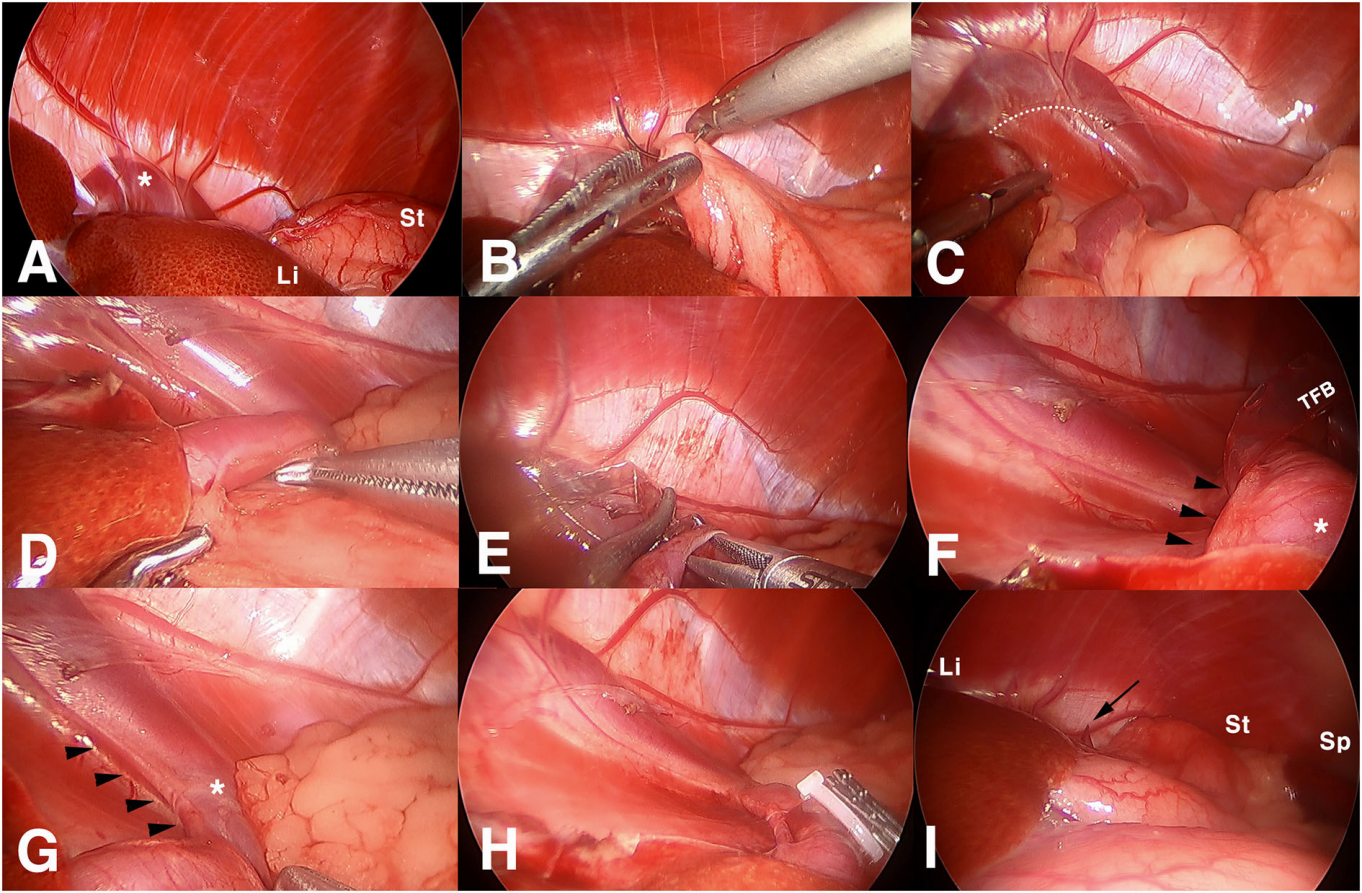
Surgical images of laparoscopic attenuation of canine splenophrenic shunt using thin-film banding. **(A)** Laparoscopic view of diaphragm in left upper abdomen, **(B)** a suture being placed to the stomach for marionette retraction, **(C)** a shunting vessel entering the diaphragm, **(D)** dissecting shunting vessel from surrounding fat, **(E)** thin film band being passed through a perivascular window, **(F)** an arterial vessel identified inside the loop of thin film banding, **(G)** phrenic artery running alongside the shunting vessel, **(H)** completed thin film banding after additional intervascular dissection, and **(I)** final view after intraabdominal organs restored their position. Asterisk, shunting vessel; Li, liver; St, stomach; dotted line, incised line of the triangular ligament; TFB, thin-film band; arrowheads, arterial branch heading to the diaphragm; black arrow, the tip of the TFB; Sp, spleen.

When the upper abdomen was explored, a portion of the shunting vessel was identified in the tendinous center of the diaphragm, covered with a left lateral liver lobe (Figure 3A). A left triangular ligament of the liver was incised using a j-hook and scissor, and the stomach was retracted to the left and caudally. A 3-0 polydioxanone suture, with a tapered point and round bodied needle, was introduced through the port, and a stay suture was placed at gastric fundus near the lesser curvature, tied intracorporeally (Figure 3B). The needle was retrieved caudolateral to the third port through the abdominal wall percutaneously, and the suture was retracted in appropriate tension to expose the area of the lesser curvature. It was fixed with a mosquito clamp extracorporeally. It was identified that a shunting vessel was running along the lesser curvature, making a flexure before entering the diaphragm (Figure 3C).

After laparoscopic visualization was secured, the shunting vessel was dissected using Maryland forceps and right angle forceps before the flexure (Figure 3D). A window was made from the dorsolateral to the medial side of the vessel. An ethylene oxide-sterilized thin-film band, folded in three layers (10 cm long and 5 mm in width), was introduced *via* the port, and it was passed through the window (Figure 3E). When the band was wrapped around the shunt and a fixation clip was about to be applied, arterial pulsation was identified inside the loop (Figure 3F). The initial band was removed, and the arterial vessel was found to be running alongside the dorsomedial aspect of the shunting vessel to the diaphragm (Figure 3G). An additional dissection was performed between them to separate the shunt from the artery. Subsequently, the second TFB was applied around the shunt only. Exercising a caution not to squeeze the diameter of the shunt, a medium-sized polymer locking ligation clip (PLLC; Hem-o-Lok^®^, Weck polymer ligation System, USA) was applied first, and three more titanium ligation clips (TLCs) were applied distally to the primary PLLC (Figure 3H). The excess band was transected at 1 cm distal to the clips and removed accordingly. After the stay suture was released, the liver and stomach were restored to their location (Figure 3I).

When the abdominal cavity was re-explored, there was no change in color or mobility of the small intestines and pancreas. Bleeding was minimal throughout the procedure. Portal sites were closed routinely with a bupivacaine infiltration. Under stable anesthesia, a cystolithotomy was performed *via* a 2 cm mini-laparotomy on the right caudolateral to the prepuce (Figure 2B), and 98 urinary stones with 1~4 mm in diameter were removed. A liver biopsy and stone analysis were not consented to by the owner. Based on the surgical finding, CTA images were re-evaluated, and the concurrent running vessel beside the shunting vessel was suspected to be a phrenic artery, which originated from the celiac artery, and was inserted into the diaphragm.

The patient recovered uneventfully and showed good appetite and normal activity immediately. He showed jumping or ground digging and no painful expressions (tenderness, flinching, whimpering, and biting) on abdominal palpation. However, the dog was hospitalized for the concern of developing post-attenuation neurologic signs. He was discharged on postoperative day 4 with the same oral medication except for levetiracetam. The patient was followed up regularly, and he was doing great with normal urination. The owner reported considerably decreased frequency of vomiting; intermittent vomiting remained only with a long fasting condition. Blood parameters of BUN, albumin, glucose, cholesterol, and fasting ammonia were maintained within the normal range (Table 1), but bile acid levels were high, without any suspected liver pathology, in diagnostic imaging. The portal vein diameter increased to 5.1 mm at 3 months after surgery (Pv/Ao ratio: 0.72). According to the owners, the patient became less aggressive and less edgy after surgery. Oral medications and lactulose were discontinued one by one 1 month after surgery, and the patient was introduced to a normal diet at 8 months after surgery.

At 14 months after shunt attenuation, a CTA was performed because of the splenic nodule grown up to 2 cm with splenic lymph node enlargement. It demonstrated a gap of contrast discontinuity about 1 cm around the clips on the same plane as in preoperative CTA, which was interpreted as a complete closure of the shunting vessel (Figures 1C,D). Before and after the gap, the vessel diameter was measured to be up to 4.6 mm, and there was no evidence of acquired shunt. When the liver volume was evaluated at this point, it was 190 cm^3^, which was 94 cm^3^ on preoperative CTA. The phrenic artery was intact, and the Pv/Ao ratio at this time was 0.75. No remarkable findings were identified in liver parenchyma on both CTA and ultrasonography, and hematologic liver panels were within reference range. Some of the nephroliths had moved to the bladder; however, it induced no clinical signs in urination. A diagnosis of indolent lymphoma was made on histopathology after splenectomy; a portovenography was not performed during the surgery. The patient was doing well for 31 months after shunt attenuation, enjoying treats without protein restriction.

## Discussion

Splenophrenic shunt drains into the CVC caudally to the diaphragm and cranially to the liver, and its shunting blood flow is readily collapsed by the diaphragm during respiratory cycles and easily compressed by a full stomach after eating (2). This anatomic structure attributes to low shunt fraction and preserved portal vein size, which is related to its clinical features to be diagnosed at a significantly older age with less severe clinical signs (median age of 51.5 vs. 6 months in the splenocaval type shunt) (4). Although portocaval shunts have been dominant in CEPSSs (64–80%) (5), a recent study of 172 canine CEPSSs reported splenophrenic shunts as the most frequent type (37.2%), followed by the splenoazygos, right gastrocaval, and splenocaval shunts (4). The patient in this report was also diagnosed belatedly despite his abnormal values of BUN, fasting ammonia, bile acids, and low Pv/Ao ratio. A chronic course of urolithiasis induced symptoms intermittently; however, the owner did not feel the necessity of urgent clinical intervention.

Generally speaking, occluding devices in CEPSS attenuation are placed close to the CVC (12). However, in splenophrenic shunts, it is recommended to perform the vessel isolation and placement of TFB or ARC before the shunt enters the diaphragm (13). On the central tendon of the diaphragm, it is not easy to dissect the shunt exclusively without the tendon fiber, which attributes to occlusion failure despite an adequate inflammatory response. Laparoscopy, in this case, provided a superior visualization up to the extreme upper and dorsal cavity of the abdomen to apply TFB before the diaphragm. However, gravity was not enough to retract the stomach even in the reverse Trendelenburg position; a stay suture was useful to expose the target structure in the deep area of lesser curvature, creating a sufficient working space. However, either the triangular ligament incision or gastric stay suture would not always be compulsory, or otherwise, several sutures of marionette technique (14) also can be placed if needed; it could be performed by case-based decision.

In addition to lack of tactile feedback and higher medical cost, laparoscopic procedures may have potential risks associated with capnoperitoneum; excessive intra-abdominal pressure may impair the venous return, and the compression of the diaphragm could result in respiratory compromise (12, 15), but these concerns could be avoided under low intra-abdominal pressure (6–10 mmHg) (12). Moreover, it causes significantly lower surgical stress in dogs, resulting in lower pain score, subsequent minimal decrease in postsurgical activity, and short hospitalization in comparison with open surgery (15, 16). In this case, the patient was discharged on postoperative day 4 to monitor development of postoperative neurologic signs. So, the duration of the hospitalization could not have been different, even if we did choose PSS attenuation with open surgery. However, the degree of pain and healing process that the patient experienced would have been quite different; he restored excellent appetite and vigorous activity on the day of surgery. As for laparoscopic PSS attenuation handling vascular structure, inability to locate the vessel, misidentification of the shunting vessel, or hemorrhage with inadvertent tearing of the vessel could be great concerns (10). However, it would not be that difficult to find and to maneuver the shunt for experienced surgeons who are familiar with PSS attenuation under open surgery, and preoperative CTA provides enough information about anatomic structure of the shunt. In this case, a great magnified visualization was also helpful to preserve the additional vascular structure, phrenic artery, which was not remarkably focused on in the CTA interpretation before surgery. A thorough investigation of preoperative examinations is mandatory; an intraoperative exploration also should not be overlooked.

A recent meta-analysis study reported a statistically significant superiority of ARC over TFB in shunt closure with an odds ratio of 36.58 (17). However, there was no statistical difference in the perioperative and the clinical outcomes; success rates of perioperative, clinical, and surgical outcomes in ARC and TFB were 94.5, 94.6, and 82.0% and 96.7, 97.8, and 56.7%, respectively. With regard to continued shunting, ARCs are considered to be more reliable than TFB (31%); however, Falls et al. reported that the persistent shunting with ARC is 24.1%, which included development of multiple acquired shunts, too (8, 9, 18). In the present case, TFB was selected for a large shunting vessel about 10 mm because TFB is adjustable regardless of the vessel diameter. It is also easily introduced through the port and has no concerns about premature closure occurring with ARC occasionally that results from vascular kinking or collapse due to the weight of an implant (10, 12, 17). Although the author has been using TFBs in laparoscopic PSS attenuations, ARCs could be used in laparoscopic attenuation; however, there would be a need to use a larger port for ARC introduction and dropping ARC device or key in the body cavity could be bothersome during placement sometimes (19). Moreover, a tendency was reported that ARCs in larger diameters are at risk of incomplete occlusion (20). Each technique has pros and cons, which could be selected based on individual case.

TFB in PSS attenuation induces an inflammatory reaction (like a chronic foreign body), which leads to thrombus formation and gradual attenuation of the shunting vessel (3). In general, TFB takes 8 weeks to close the shunt; however, there has been inconsistency concerning the long-term surgical outcome, which is attributed to factors like diameter of the shunting vessel, degree of intraoperative attenuation performed by surgeons, and material composition of TFB (3, 17). TFBs that have been used for PSS attenuation vary in composition and molecular structure from medical grade pure cellulose-based cellophane to non-medical polypropylene (3, 11). A recent study reported that non-cellulose-based TFB (polyolefin/polypropylene fiber) resulted in similar surgical outcomes for gradual attenuation of a single CEHPSS in comparison with ARC with low postoperative morbidity in short- and long-term follow-up (3). TFB, applied in the present case without intraoperative attenuation for a large shunt, was a non-medical, commercial cellophane wrapper which induced complete closure of the shunt. Although an intraoperative attenuation to make the shunt diameter less than 3 mm during TFB used to be recommended, TFB without intraoperative attenuation had been found to be also effective (21). In the latest study of 20 canine laparoscopic CEPSS attenuations using TFB with intraoperative partial occlusion (50%) (11), four dogs (20%) experienced postoperative portal hypertension; three of them recovered with conservative therapy, but the remaining needed a revision surgery with severe clinical signs. Although conversion to open surgery was required in five dogs (25%), 19 dogs (95%) had a good outcome showing resolution of clinical signs within 2 months, consequently.

As in this case, TFBs are usually placed in three folds and secured using 3–4 metallic hemoclips (3, 11, 12), but Joffe et al. recommended a four-layer cellophane band secured with a single medium-sized PLLC or TLC with a minimal failure in a recent study (22). If postoperative CTA is planned, PLLC would be better as it is inert, non-conductive, compatible with CT or MRI. It also provides good security with the lock engagement feature of teeth in the jaws (23).

Various methods have been used to estimate surgical outcomes after CEPSS attenuation. Serum bile acid concentration, especially measured post-prandial, is a reliable parameter to diagnose PSS with 100% of sensitivity and specificity; however, it is not a prognostic indicator to determine surgical success (24). It is not uncommon that it gives false negative for persistent residual shunting or the development of multiple acquired PSS and false positive for complete closure as well (25). This is also not that different in the case of fasting ammonia concentration. Currently, none of the laboratory parameters alone can play a role in confirming the complete closure of the shunt. Therefore, clinical outcomes, liver function tests, and diagnostic imaging should be combined to evaluate the PSS closure. Fasting ammonia level in this patient returned to within the normal range; however, the bile acids remained high in both the pre- and post-prandial tests regardless of improvement in clinical signs and blood examination (BUN, albumin, and cholesterol).

A previous case report of canine portocaval shunt attenuation using TFB described a change in liver size showing a normal hepatic ratio (a value obtained by dividing the liver length by the length of the second lumbar vertebral body on a lateral radiograph) at 10 days after surgery (26). However, liver size in radiographic images can be different according to direction of lateral recumbency and this positional effect can alter the result occasionally (27). As for Pv/Ao ratio, an ultrasonographic study reported that the ratio was less than 0.65 in patients with CEPSS, whereas dogs and cats with a ratio greater than 0.8 did not have the disease (28). Pv/Ao can be helpful occasionally to diagnose CEPSS. However, it cannot be applied similarly to all types of CEPSS, which was also mentioned in a recent report that showed a ratio of 0.83 as the mean Pv/Ao ratio in 64 splenophrenic shunts (4). Moreover, during the post-attenuation follow-up in the present case, the increase of portal vein diameter in absolute value (from 3 mm to 5.3 mm) or Pv/Ao ratio (from 0.42 to 0.75) implied an increasing tendency of portal perfusion, but it could not ascertain a complete closure of the shunt.

Diagnostic imaging techniques (portovenography, CTA, portal scintigraphy, abdominal ultrasonography, and magnetic resonance angiography) have also been used for the evaluation of shunt closure. However, the timing of postoperative imaging has not been standardized (17). Even though general anesthesia is required, the CTA is non-invasive, intuitive, and confirmative for determining the complete occlusion of the shunt, investigating acquired post-attenuation PSS formation, and evaluating hepatic volume expansion subsequent to surgery in quantitative measurement. It can also give information about diameter or location of persistent shunts when a revision surgery is needed. Postoperative CTA demonstrated a complete transection of vascular blood continuity, no additional shunt development, and more than two times of hepatic volume expansion in the present case. Recently, measurement of serum hyaluronic acid concentration or injection of lidocaine and measurement of its metabolite, monoethylglycinexylidide, have been proposed as a promising, non-invasive biomarker to determine shunt closure and liver perfusion, and these tests do not require anesthesia (29, 30).

## Conclusion

This is a case of laparoscopic attenuation of congenital extrahepatic splenophrenic shunt in a dog using a TFB without intraoperative attenuation. Laparoscopy provided optimal visualization and sufficient working space with stay suture and table tilting throughout the procedure, and there was no need of conversion. With a minimal hemorrhage, there were no perioperative or long-term complications. A favorable surgical outcome of complete closure of the shunt with liver volume expansion was confirmed with post-attenuation CTA. The patient was alive at 31 months after surgery without any clinical disturbances of neurologic and urinary symptoms. The findings in this case report supported that laparoscopic attenuation is clinically practical for splenophrenic shunt in small-breed dogs. More clinical trials for further data accumulation are warranted in many clinical patients with long-term follow-up periods in future.

## Data availability statement

The original contributions presented in the study are included in the article/supplementary material, further inquiries can be directed to the corresponding author.

## Author contributions

JP collected the data and wrote the first draft of the manuscript. KJ and HJ supported the data collection and participated in editing the manuscript. SK reviewed and edited the manuscript. All authors contributed to the manuscript and approved the manuscript submission.

## Funding

This work was supported by Korea Institute of Planning and Evaluation for Technology in Food, Agriculture and Forestry (IPET) through Development and service establishment of cell therapy for incurable stomatitis in companion animals using stem cells Project, funded by Ministry of Agriculture, Food and Rural Affairs (MAFRA) (grant number 322097-03-1-CG000).

## Conflict of interest

The authors declare that the research was conducted in the absence of any commercial or financial relationships that could be construed as a potential conflict of interest.

## Publisher's note

All claims expressed in this article are solely those of the authors and do not necessarily represent those of their affiliated organizations, or those of the publisher, the editors and the reviewers. Any product that may be evaluated in this article, or claim that may be made by its manufacturer, is not guaranteed or endorsed by the publisher.
